# HLA and red blood cell antigen genotyping in SARS-CoV-2 convalescent plasma donors

**DOI:** 10.2217/fvl-2022-0058

**Published:** 2023-02-20

**Authors:** William Lemieux, Josée Perreault, Gabriel André Leiva-Torres, Nadia Baillargeon, Jessica Constanzo Yanez, Marie-Claire Chevrier, Lucie Richard, Antoine Lewin, Patrick Trépanier

**Affiliations:** ^1^Héma-Québec, Medical Affairs & Innovation, Québec City & Montréal, Québec, G1V 5G3, Canada; ^2^Héma-Québec, Transfusion Medicine, Québec City & Montréal, Québec, H4R 2W7, Canada

**Keywords:** COVID-19, human leukocyte antigens, red blood cell antigens, SARS-CoV-2, susceptibility

## Abstract

**Aim:** More data is required regarding the association between HLA allele and red blood cell (RBC) antigen expression in regard to SARS-CoV-2 infection and COVID-19 susceptibility. **Methods:** ABO, RhD, 37 other RBC antigens and HLA-A, B, C, DRB1, DQB1 and DPB1 were determined using high throughput platforms in 90 Caucasian convalescent plasma donors. **Results:** The AB group was significantly increased (1.5×, p = 0.018) and some HLA alleles were found to be significantly overrepresented (HLA-B*44:02, C*05:01, DPB1*04:01, DRB1*04:01 and DRB1*07:01) or underrepresented (A*01:01, B51:01 and DPB1*04:02) in convalescent individuals compared with the local bone marrow registry population. **Conclusion:** Our study of infection-susceptible but non-hospitalized Caucasian COVID-19 patients contributes to the global understanding of host genetic factors associated with SARS-CoV-2 infection and severity.

The COVID-19 pandemic, caused by the SARS-CoV-2, has caused over 6.6 million deaths worldwide as of December 2022 (https://covid19.who.int/). Intense work has been done since the beginning of the pandemic to protect the most at-risk populations. Being male, of older age, obese, of a certain ethnic origin, having diabetes, asthma, or many other medical conditions, have all been associated with an increased risk of COVID-19-related complications or death [1–5]. Genetic factors, such as the expression of angiotensin-converting enzyme (ACE)-related genes, may also play a role in disease severity and could serve as a predictive marker for at-risk populations [6]. Efforts in getting a better understanding of the molecular gateways for viral entry and of the host's response have been relentless, focusing on ACE2 expression, HLA, cytokine storms and TLRs [7]. Such research could lead to an improved understanding of the disease's susceptibility and severity, the capacity for virus clearance, long term symptoms [8] and mortality consequent to infection in humans.

The relationship between ABO blood group expression and susceptibility to infection by SARS-CoV-2 have been explored by a few groups and recently reviewed [9,10]. Group O individuals were identified as having a decreased risk of infection compared with other ABO groups, although no differences were observed regarding hospitalization and death rates caused by COVID-19 [11,12]. Several hypotheses to explain this observed link between ABO type and risk of infection have been suggested, such as the presence of anti-A and -B antibodies in O individuals [13] and the binding of the receptor-binding domain (RBD) of the viral Spike protein to group A antigens [14]. Given the important immunological deregulation and potential cytokine storm associated with mortality in COVID-19 patients [15] and the interesting report from an Italian laboratory of higher rates of direct antiglobulin test (DAT) reactivity in COVID-19 patients [16], RBC antigens and related antibodies could be involved in COVID-19 and its clinical manifestations.

Of additional importance is the potential contribution of HLAs, given their central role in regulating immunity against viruses [17]. Considerable efforts have been made in trying to identify protective or susceptibility-enhancing HLA alleles [18,19]. Binding assays have shown that HLA-A*02:01 and B*40:01 can preferentially associate with SARS-CoV-2 epitopes [20]. Using bioinformatic prediction, HLA-A*02:03 and A*31:01 were identified as protective, while A*03:02 was identified as a risk allele [21]. *In silico* binding affinity studies have shown that HLA-B*46:01 could increase susceptibility to disease, whereas HLA-B*15:03 is associated with protective immunity. and the same studies found that HLA-A*02:02, B*15:03, and C*12:03 were the most frequently encountered haplotypes associated with the presentation of viral epitopes [18]. Other retrospective studies aimed at identifying potentially protective or risk-increasing alleles [22–28] have been published. Such an effort requires several geographically diverse laboratories to analyze and share available data in order to generate a comprehensive overview.

In the present study, we are also advancing the hypothesis that associations might exist between certain HLA alleles or blood groups and SARS-CoV-2 susceptibility, as well as the ability to overcome infection without hospitalization. We therefore sought to analyze and determine the existence of any trends in the expression of an extended panel of RBC antigens (ABO, RhD and 37 other antigens), and of HLA-A, B, C, DRB1, DQB1 and DPB1 alleles within a Caucasian convalescent plasma donor cohort, in comparison to different reference frequencies (textbook, local and international databases, and literature). The identification of differential patterns of RBC antigen or HLA expression in convalescent individuals, who were infected but were not hospitalized, could contribute to a better understanding of SARS-CoV-2 susceptibility and COVID-19 severity.

## Materials & methods

### Samples

We analysed a cohort of 90 Caucasian convalescent plasma donors. Donors were randomly chosen from adult participants of the Québec cohort in the CONCOR-1 clinical trial (#NCT04348656). All subjects received an official diagnosis of COVID-19 by the Québec Provincial Health Authority after epidemiologic investigation or after confirmation by polymerase chain reaction (PCR) test. All subjects were COVID-19 symptomatic during infection, cleared the infection without hospitalization, and were free of symptoms for at least two weeks before donating. Since COVID-19 diagnostic was given before spring 2021, subjects were presumably infected with the Alpha (B.1.1.7) or Beta (B.1.351) variant, since no case of the Delta (B.1.617.2) variant had yet been reported in the province of Quebec [29]. All subjects were self-identified Caucasians. Our convalescent plasma donor cohort had an average age of 40.4 ± 15.0 years and consisted of 68% males. Donors were not selected with respect to their ABO group. All donors gave consent to participate in this research project, which was approved by the Héma-Québec Research Ethics Committee. Control populations were from the National Marrow Donor Program (NMDP) registry and from Héma-Québec's bone marrow donor registry. For the Héma-Québec control cohort, 1370 registered individuals typed in high-resolution, from the Montréal and Montérégie regions and of self-reported Caucasian ethnicity, were selected to match with the characteristics of the studied convalescent cohort. No variable other than geographical region and ethnicity was matched, and all donors were eligible to donate plasma in Québec.

### Phenotyping & genotyping

ABO and RhD phenotype testing was done by serologic detection using the PK7300 from Beckman Coulter, as per the manufacturer's protocol. DNA used for genotyping was extracted from the buffy coat of whole blood samples collected in ethylenediaminetetraacetic acid (EDTA) tubes, using QIAamp Blood Mini kit (Qiagen, Hilden, Germany). RBC genotyping was performed using the Luminex xMAP^®^ technology with the ID CORE XT platform (Progenika Biopharma-Grifols, Bizkaia, Spain), as per the manufacturer's protocol, for the following blood group antigens: Rh (C, c, E, e, C^w^, hr^S^, hr^B^, V, VS), Kell (K, k, Kp^a^, Kp^b^, Js^a^, Js^b^), Kidd (Jk^a^, Jk^b^), Duffy (Fy^a^, Fy^b^), MNS (M, N, S, s, U, Mi^a^), Diego (Di^a^, Di^b^), Dombrock (Do^a^, Do^b^, Hy, Jo^a^), Colton (Co^a^, Co^b^), Yt (Yt^a^, Yt^b^) and Lutheran (Lu^a^, Lu^b^). HLA genotyping was done by next-generation sequencing (NGS) on a MiSeqDx (Illumina, CA, USA), using NGSgo^®^-Ampx v2 kits, and interpreted with NGSengine^®^ v2.21 (both from GenDX, Utrecht, The Netherlands).

### Statistical analyses

For RBC antigen and HLA allele comparisons in Tables 1–3, population-wide proportions were assumed to correspond to the Caucasian prevalence estimates (from the Blood Group Antigen FactsBook [30] and from the National Marrow Donor Program database [31]), while the HLA G-group allele frequencies of the 90 participants (180 individual HLA alleles) were estimated along with the Clopper-Pearson 95% confidence interval. Z-tests for two proportions were used to test for statistical significance between populational and observed antigen prevalence. A p value inferior to the Bonferroni correction for multiple comparison per antigen group was considered significant.

**Table 1.** RBC antigen frequencies for the Caucasian convalescent plasma donors compared with the Factsbook reference frequencies.

Blood group system	Predicted phenotype	Observed prevalence	CI 95%	FactsBook prevalence	p-value	Post-test p-value cutoff
Rh	D- C-c+E-e+	0.156	[0.095–0.244]	0.151	0.897	0.0045
	D- C-c+E+e+	0.022	[0.006–0.077]	0.009	0.194	
	D+ C-c+E+e+	0.100	[0.054–0.179]	0.118	0.596	
	D+ C+c-E-e+	0.233	[0.158–0.331]	0.185	0.242	
	D+ C+c+E-e+	0.322	[0.235–0.424]	0.349	0.589	
	D+ C+c+E+e+	0.167	[0.104–0.257]	0.133	0.342	
	Cw	0.011	[0.002–0.060]	0.020	0.478	
	hrS	1.000	[0.959–1.000]	0.980	0.174	
	hrB	1.000	[0.959–1.000]	0.980	0.174	
	V	0.000	[0.000–0.041]	0.010	0.342	
	VS	0.000	[0.000–0.041]	0.000	1.000	
Kell	K+k-	0.000	[0.000–0.041]	0.002	0.675	0.006
	K-k+	0.944	[0.876–0.976]	0.910	0.259	
	K+k+	0.056	[0.024–0.124]	0.088	0.286	
	Kp(a+b-)	0.000	[0.000–0.041]	0.000	1.000	
	Kp(a-b+)	0.956	[0.891–0.983]	0.977	0.184	
	Kp(a+b+)	0.044	[0.017–0.109]	0.023	0.184	
	Js(a+b-)	0.000	[0.000–0.041]	0.000	1.000	
	Js(a-b+)	1.000	[0.959–1.000]	1.000	1.000	
	Js(a+b+)	0.000	[0.000–0.041]	0.000	1.000	
MNS	M+N+S+s+	0.267	[0.186–0.366]	0.240	0.549	0.005
	M+N+S-s+	0.144	[0.086–0.232]	0.220	0.082	
	M-N+S-s+	0.111	[0.061–0.193]	0.150	0.298	
	M+N-S+s+	0.178	[0.112–0.269]	0.140	0.298	
	M+N-S-s+	0.122	[0.070–0.206]	0.080	0.142	
	M-N+S+s+	0.078	[0.038–0.152]	0.060	0.472	
	M+N-S+s-	0.033	[0.011–0.093]	0.060	0.280	
	M+N+S+s-	0.067	[0.031–0.138]	0.040	0.190	
	U	1.000	[0.959–1.000]	0.999	0.764	
	Mia	0.000	[0.000–0.041]	0.000	1.000	
Duffy	Fy(a+b-)	0.256	[0.177–0.354]	0.170	0.030	0.017
	Fy(a-b+)	0.344	[0.254–0.447]	0.340	0.936	
	Fy(a+b+)	0.400	[0.305–0.503]	0.490	0.087	
	Fy(a-b-)	0.000	[0.000–0.041]	Very rare	N/A	
Kidd	Jk(a+b-)	0.344	[0.254–0.447]	0.263	0.082	0.017
	Jk(a-b+)	0.222	[0.149–0.318]	0.234	0.787	
	Jk(a+b+)	0.433	[0.336–0.536]	0.503	0.184	
	Jk(a-b-)	0.000	[0.000–0.041]	Rare	N/A	
Diego	Di(a+b-)	0.000	[0.000–0.041]	0.000	1.000	0.017
	Di(a-b+)	1.000	[0.959–1.000]	0.999	0.764	
	Di(a+b+)	0.000	[0.000–0.041]	0.001	0.764	
Dombrock	Do(a+b-)	0.133	[0.078–0.219]	0.180	0.246	0.010
	Do(a+b+)	0.500	[0.399–0.601]	0.490	0.849	
	Do(a-b+)	0.367	[0.274–0.470]	0.330	0.453	
	Hy-	0.000	[0.000–0.041]	0.000	1.000	
	Jo(a-)	0.000	[0.000–0.041]	0.000	1.000	
Colton	Co(a+b-)	0.900	[0.824–0.946]	0.900	1.000	0.013
	Co(a-b+)	0.000	[0.000–0.041]	0.005	0.503	
	Co(a+b+)	0.100	[0.054–0.179]	0.095	0.873	
	Co(a-b-)	0.000	[0.000–0.041]	0.000	1.000	
Yt	Yt(a+b-)	0.889	[0.807–0.939]	0.919	0.298	0.017
	Yt(a+b+)	0.111	[0.061–0.193]	0.078	0.242	
	Yt(a-b+)	0.000	[0.000–0.041]	0.003	0.603	
Lutheran	Lu(a+b-)	0.000	[0.000–0.041]	0.002	0.675	0.0125
	Lu(a-b+)	0.922	[0.848–0.962]	0.924	0.944	
	Lu(a+b+)	0.078	[0.038–0.152]	0.074	0.889	
	Lu(a-b-)	0.000	[0.000–0.041]	0.000	1.000	

**Table 2.** ABO and RhD blood group distributions for the Caucasian convalescent plasma donors compared with Factsbook reference frequencies.

Group	Antigen	Observed prevalence	CI 95%	FactsBook prevalence	p-value^†^
All donors (n = 90)	A	0.456	[0.357–0.558]	0.430	0.569
	AB	0.089	[0.046–0.166]	0.040	**0.018**
	B	0.100	[0.054–0.179]	0.090	0.741
	O	0.356	[0.264–0.458]	0.440	0.110
	D-	0.178	[0.112–0.269]	0.150	0.459
	D+	0.822	[0.731–0.888]	0.850	0.459
FY*A/*A individuals (n = 23)	A	0.435	[0.256–0.632]	0.430	0.960
	AB	0.130	[0.045–0.321]	0.040	**0.028**
	B	0.130	[0.045–0.321]	0.090	0.503
	O	0.304	[0.156–0.509]	0.440	0.190

† Bold p values represents a significant p value of <0.05.

**Table 3.** Caucasian convalescent donor HLA allele frequency comparison with the NMDP database for HLA*A, B, C, DRB1 and DQB1. The g notation specifies G-groups corresponding to those presented in Gragert, Loren, Abeer Madbouly, John Freeman, and Martin Maiers. Six-locus high resolution HLA haplotype frequencies derived from mixed-resolution DNA typing for the entire US donor registry. *Human Immunology* 74, no. 10.

Loci	Alleles	Observed frequency (2n = 180)	CI 95%	NMDP frequency	Adjusted p-value
HLA-A	02:01G	0,267	[0.204–0.338]	0,272	1,000
	01:01G	0,122	[0.078–0.179]	0,159	1,000
	03:01G	0,117	[0.074–0.173]	0,140	1,000
	24:02G	0,072	[0.039–0.120]	0,089	1,000
	29:02G	0,061	[0.031–0.107]	0,031	0,427
	11:01G	0,061	[0.031–0.107]	0,059	1,000
HLA-B	44:02G	0,128	[0.083–0.186]	0,087	1,000
	07:02G	0,089	[0.052–0.140]	0,125	1,000
	08:01G	0,083	[0.047–0.134]	0,106	1,000
	44:03	0,072	[0.039–0.120]	0,044	1,000
	40:01G	0,067	[0.035–0.114]	0,050	1,000
	18:01G	0,061	[0.031–0.107]	0,047	1,000
	35:01G	0,061	[0.031–0.107]	0,058	1,000
	27:05G	0,056	[0.027–0.100]	0,035	1,000
HLA-C	05:01G	0,1389	[0.092–0.198]	0,083	0,109
	04:01G	0,1222	[0.078–0.179]	0,115	1,000
	07:01G	0,1167	[0.078–0.179]	0,155	1,000
	06:02G	0,0944	[0.056–0.147]	0,095	1,000
	07:02G	0,0944	[0.056–0.147]	0,137	1,000
	02:02G	0,0889	[0.052–0.140]	0,047	0,138
	12:03G	0,0722	[0.039–0.120]	0,056	1,000
	03:04G	0,0667	[0.035–0.114]	0,074	1,000
HLA-DRB1	07:01	0,178	[0.125–0.242]	0,130	1,000
	01:01	0,100	[0.060–0.153]	0,085	1,000
	03:01	0,089	[0.052–0.140]	0,114	1,000
	04:01	0,089	[0.052–0.140]	0,080	1,000
	13:01g	0,083	[0.047–0.134]	0,000	0,000
	15:01	0,078	[0.043–0.127]	0,129	0,985
	11:01g	0,056	[0.027–0.100]	0,063	1,000
	14:01g	0,056	[0.027–0.100]	0,027	0,497
HLA-DQB1	02:01g	0,211	[0.154–0.278]	0,212	1,000
	03:01g	0,200	[0.144–0.266]	0,196	1,000
	05:01	0,133	[0.087–0.192]	0,116	1,000
	06:03g	0,089	[0.052–0.140]	0,065	1,000
	06:02	0,078	[0.043–0.127]	0,128	0,621
	03:02g	0,067	[0.035–0.114]	0,103	1,000
	03:03g	0,056	[0.027–0.100]	0,043	1,000
	05:03g	0,056	[0.027–0.100]	0,029	0,407

Alleles with less than 10 data points are not shown.

NDMP: National Marrow Donor Program.

Allele frequencies were calculated using the GENE[RATE] tool for HLA-A, B, C, DRB1, DQB1, and DPB1 [32]. HLA allelic frequencies were used with a modification of the hierfstat package to calculate the genetic distance (latter) globally and for each locus between the subjects and the reference population [33,34]. Standardized residuals of the cohort subjects were calculated to identify alleles with significant differences between the subjects and the registry. Residuals were calculated by considering the subjects' allele frequencies as the independent variable and the registry frequency as the dependant variable for each locus independently. Frequencies were deemed different at or above a difference of 3 absolute standardized residual. Statistical analyses were performed using R [35].

## Results

### Red blood cell genotype frequencies

Genotype frequencies for Rh, Kell, MNS, Duffy, Kidd, Diego, Dombrock, Colton, Yt and Lutheran blood groups were determined in each individual, and the resulting predicted phenotypes were compared with the expected Caucasian reference frequencies. The FY*A/*A genotype (Fy[a+b-] predicted phenotype) appears to be overrepresented (nonsignificant, p = 0.030) in our convalescent cohort compared with expected frequencies (0.256 vs 0.170, respectively), as presented in Table 1. Incidentally, FY*A/*B (Fy[a+b+] predicted phenotype) individuals appear to be trending toward a decreased frequency of 0.400 compared with the expected 0.490 (Table 1), although the trend is not significant (p = 0.087). Overall, no antigen group combinations deviated significantly from the expected frequencies.

### ABO & RhD phenotyping

ABO and RhD phenotypes were determined for each convalescent individual and compared with the expected Caucasian reference frequencies for the entire cohort (n = 90) and within FY*A/A individuals (n = 23), given its trend toward overrepresentation (Table 1). Table 2 presents the ABO phenotyping analysis for the cohort, which allowed for the identification of a significant (p = 0.0178) 2.2× increase for the AB group compared with reference frequencies (0.089 vs 0.040, respectively). While nonsignificant (p = 0.110), the O group trends toward a 0.8× underrepresentation within convalescent individuals compared with expected frequencies (0.356 vs 0.440, respectively). Interestingly, a significant (p = 0.028) 3.3× AB group overrepresentation was also found within FY*A individuals (Table 2, FY*A/*A). An apparent but nonsignificant decrease in O group individuals can be observed within the FY*A/*A individuals versus the reference frequency (0.304 vs 0.440). No other trend or significant observation was made regarding A and B groups, or RhD expression.

### HLA allele frequency comparisons

**Figure 1.**
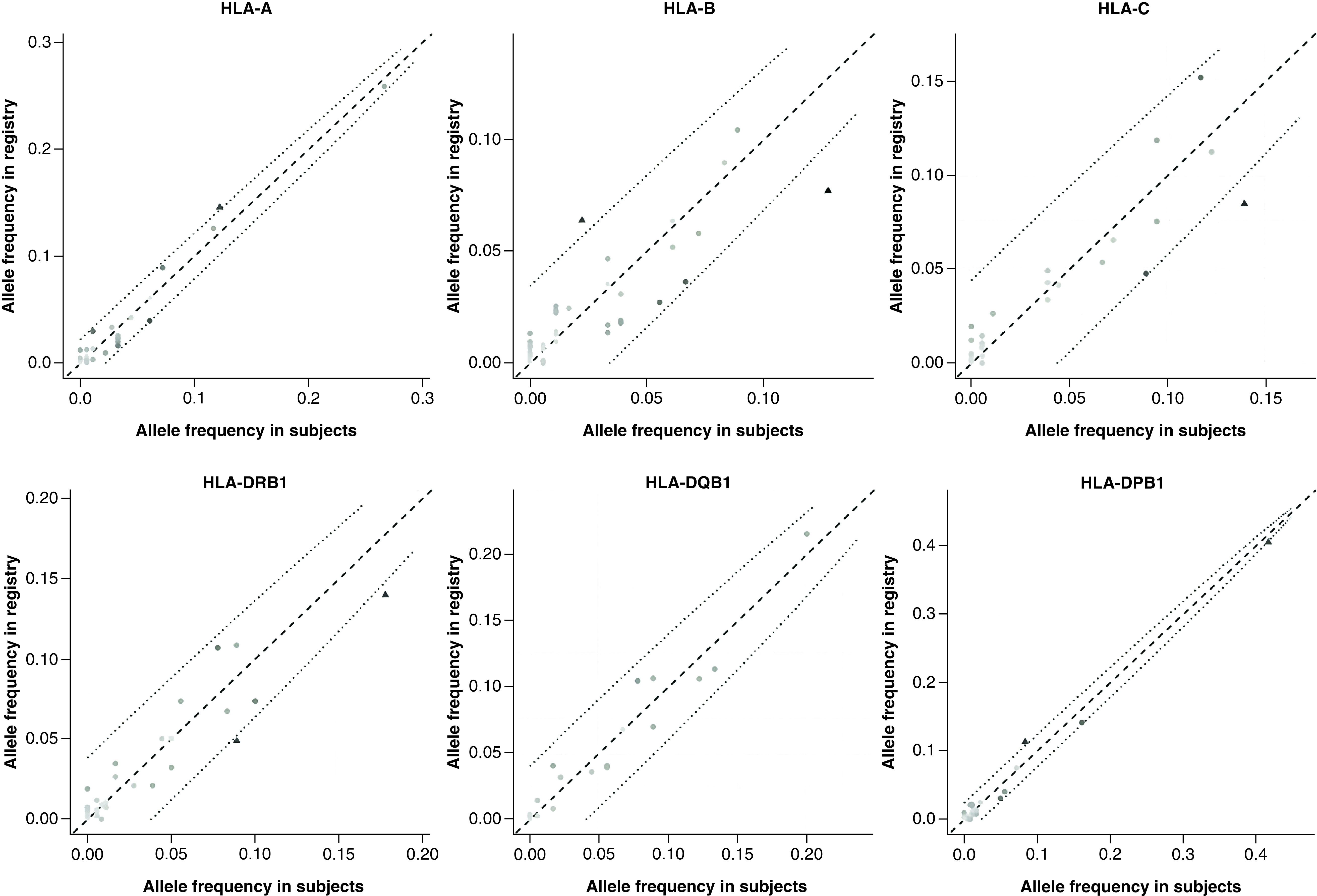
HLA Allelic frequencies in the convalescent cohort and the Héma-Québec bone marrow donor registry. For each locus, the cohort allele frequencies (horizontal axis) are plotted against the registry frequencies (vertical axis). The dashed line represents identical expression in the cohort and registry. The dotted lines are the thresholds of 3 |standardised residuals| away from the identity line.

HLA typing was done by NGS in the Caucasian convalescent donors' cohort and individual allele frequencies were determined. The convalescent cohort allele frequencies (2n = 180) were first compared with the NMDP registry frequencies for HLA-A, B, C, DRB1 and DQB1 for the most frequent alleles (Table 3), and no significant differences were identified in the most common alleles in the cohort. The convalescent cohort frequencies for HLA-A, B, C, DRB1, DQB1 and DPB1 were then compared with the Héma-Québec Stem Cell Donor Registry frequencies for Caucasians in the same geographical region (2n = 2740). The genetic distance between the convalescent donors' cohort and the Héma-Québec Registry was calculated for all loci together and on a per-locus basis for HLA-A, B, C, DRB1, DQB1, and DPB1 (Table 4). There was no comparative measurement available for the genetic distance, however the distance is low in all loci compared. Standardized residual analysis was conducted (Table 5 & Figure 1) and led to the identification of alleles that were significantly different between the convalescent cohort (n = 90 individuals) and the Héma-Québec Registry (n = 1370 individuals). HLA-B*44:02, C*05:01, DPB1*04:01, DRB1*04:01 and DRB1*07:01 were significantly overrepresented within our cohort, while A*01:01, B51:01 and DPB1*04:02 were significantly underrepresented. Finally, the alleles identified as significantly different were used to search previously published data for suspected associations with the SARS-CoV-2 virus infection and COVID-19 disease characteristics; these results are presented in Table 5. Of note, for two of the eight alleles that we have identified (DPB1*04:02 and DRB1*07:01), there was no available data in the literature for comparison.

**Table 4.** Genetic distance comparison between Caucasian convalescent donor HLA allele frequencies (2n = 180) and Héma-Québec bone marrow donor registry (2n = 2740) for HLA*A, B, C, DPB1, DQB1 and DRB1, per loci, and globally.

Locus	A	B	C	DPB1	DQB1	DRB1	Global
Genetic Distance	0,0017	0,0050	0,0045	0,0018	0,0021	0,0040	0.0033

**Table 5.** HLA alleles identified as significantly over- or underrepresented from pairwise comparisons between the convalescent cohort (2n = 180) and Héma-Québec bone marrow donor registry (2n = 2740), and their potential clinical significance.

Alleles	Frequency		Standardised residuals^†^	Significance and references	Ref.
	Cohort	Registry			
A*01:01	0,1222	0,1456	-3,3570539	Associated with high risk, mortality	[36,37]
B*44:02	0,1278	0,0772	5,15830039	B*44 More susceptible to infection	[26]
B*51:01	0,0222	0,0640	-3,6358415	B*51 nonsignificantly correlates with infection susceptibility	[26]
C*05:01	0,1389	0,0848	4,11569996	Risk of death	[27]
DPB1*04:01	0,4167	0,4048	3,39285576	Direct association with severity	[38]
DPB1*04:02	0,0833	0,1128	-3,7313073	No data reported	
DRB1*04:01	0,0889	0,0488	3,26507272	Increased in severe vs asymptomatic	[28]
DRB1*07:01	0,1778	0,1397	3,85103473	No data reported	

† Standardised residuals ≥3 or ≤3 are considered significant.

## Discussion

Our study took a deeper look into the RBC and HLA characteristics of a COVID-19 recovered and non-hospitalized cohort enrolled in the CONCOR-1 convalescent plasma study. We used ABO and RhD automated blood donor testing, and RBC and HLA high throughput genotyping platforms to determine the existence of potential trends regarding the frequencies of ABO, RhD, and 37 other RBC antigens and HLA genotypes within the cohort, compared with reference Caucasian populations from textbooks, public databases, and our local bone marrow donor registry. A significant AB blood group overrepresentation was identified, as well as a nonsignificant trend in FY*A/A individuals. These results suggest a possible involvement of ABO and Duffy red blood cell antigens in SARS-CoV-2 susceptibility and COVID-19 severity, as all these individuals contracted the virus, yet only had mild symptoms, and cleared the infection without needing hospitalization.

The case for HLA association with disease susceptibility and severity is more complex. Overall, the genetic distance calculated from HLA allele frequencies is low (below 0.01) and suggests the cohort is similar to the reference population chosen. When looking at allele-level frequencies, eight HLA alleles were identified by the standardized residuals analysis as potential markers between the convalescent cohort and a stem cell registry from the same geographical region.

One of the limitations of this study concerns the lack of a more diverse stratification of disease severity and the limited sample size for COVID-19-affected individuals. Indeed, our study lacks blood group and HLA data from hospitalized, deceased and asymptomatic COVID-19 patients, which would be of interest given that there could be a significant link between ABO and severity [39]. Additionally, while the historical ABO frequencies of Quebec Caucasian blood donors (internal data) matched that of FactsBook, such unbiased information about the frequencies of other RBC antigens is not currently available, hence the use of FactsBook Caucasian reference frequencies. Our study also does not directly address the major RBC and HLA antigen frequency differences between ethnicities. While our convalescent plasma donation program reflected our donor pool [40], the low number of non-Caucasian individuals was insufficient to conduct statistical analysis, which is unfortunate given the importance of understanding the disproportionate impact of COVID-19 on minorities [5,41]. Nonetheless, the identification of a potential overrepresentation of FY*A/*A within Caucasian COVID-19 convalescent individuals and the potential implication of the Duffy blood group could have an impact on future research directions. The trend toward overrepresentation of the Fy(a+b-) predicted phenotype among our COVID-19 convalescent cohort could be explained by the absence of the Fy^b^ antigen, since no significant difference was observed when comparing Fy(a+b+) and Fy(a-b+). In individuals of African descent, the Fy(a-b-) phenotype is caused by a GATA box mutation upstream of the *FY* gene silencing Fy^b^ expression in RBCs [42]. Given that 67% of African Americans (AA) are Duffy null [43], and that Duffy null patients have an increased mortality rate from acute lung injury [4], some groups have already hypothesized a role for Duffy in COVID-19 AA individuals [43]. We therefore suggest that Duffy allele identification might be used to select individuals at-risk for COVID-19 complications for further research on associations between COVID-19 and RBC antigens. The involvement of ABO blood groups in COVID-19 has previously been described [13,44]. The mechanism underlying the association remains elusive, but could be related to circulating natural anti-A and anti-B antibodies, or a low-efficiency furin cleavage in O-group individuals [9]. A significant overrepresentation of the AB group, and the nonsignificant trend toward underrepresentation of the O group within our cohort, appear to be in agreement with other groups' suggestion that O individuals could be less susceptible to SARS-CoV-2 infection [11,12]. Our sample size does not allow us to draw conclusions as to whether individuals from the AB group are more susceptible to infection and more efficient at clearing infection without hospitalization, or whether this bias is a consequence of the trend toward underrepresentation of O individuals, who are less susceptible to infection. It would be interesting to extend our observations to larger cohort that include severely affected patients.

The involvement of HLA alleles in the disease outcome of COVID-19 patients is getting more evident [45,46]. Various HLA alleles have shown high binding affinity to SARS-CoV-2 peptides [18,47], and trends have been observed in many populations [22,23]. While our sample size is limited, eight alleles were identified as significantly different between the studied cohort and matched individuals from the Héma-Québec Stem Cell Donor Registry, for which we already had high resolution HLA information. Two of these eight HLA alleles have not previously been identified for their association with COVID-19: DPB1*04:02 and DRB1*07:01. The underrepresentation of HLA-A*01:01 is in agreement with its suggested association with high risk and mortality in COVID-19 individuals [36,37], and the overrepresentation of HLA-B*44:02 is compatible with an increased susceptibility to infection [26]. Interestingly, A*01:01, B*44:02 and B*51:01 were predicted as weak binder of SARS-CoV-2 peptides, and none of the other alleles we identified were found to be strong peptide binders [48]. Given that we found A*01:01 and B51:01 to be underrepresented and B*44:02 overrepresented in our cohort, it is difficult to establish a relationship between these data without considering complete haplotypes or other disease severity groups. The other associations are inconclusive, but should be considered in larger cohorts. Overall, more data is required from more diverse populations in order to develop a comprehensive view and a better understanding to manage emerging variants and infection waves.

Altogether, we provide additional information regarding the role of RBC antigens and HLA in SARS-CoV-2 susceptibility, and consequential COVID-19 susceptibility, severity, resolution and long-term clinical consequences. More research needs to be done to get a better understanding of potentially at-risk populations, and for the identification of molecular pathways of this virus.

## Conclusion

This study provides insights on the importance of considering the RBC and HLA antigens in regard to the susceptibility, severity, resolution and long-term clinical consequences of COVID-19. Genetic markers such as HLA could help focus our prevention efforts onto potentially more at-risk populations, and help organize vaccination strategies.

**Summary points**

- ABO, RhD, 37 other RBC antigens and HLA-A, B, C, DRB1, DQB1 and DPB1 were determined using high throughput platforms in 90 Caucasian convalescent plasma donors.
- The AB group was significantly increased in convalescent individuals and the FY*A/*A genotype was trending as overrepresented compared with the expected frequencies.
- HLA typing was performed on the convalescent donors and their allele frequencies were compared with the NMDP registry and the Héma-Québec Stem Cell Donor Registry. Standardized residual analysis identified eight alleles that were significantly different between the convalescent cohort and the Héma-Québec Registry.
- Previous literature was searched for suspected associations between the identified HLA alleles and SARS-CoV-2 virus infection and COVID-19 disease characteristics.
- Further research is needed to confirm the trends and associations observed in this study.
